# Current and Future Treatments for Classic Galactosemia

**DOI:** 10.3390/jpm11020075

**Published:** 2021-01-28

**Authors:** Britt Delnoy, Ana I. Coelho, Maria Estela Rubio-Gozalbo

**Affiliations:** 1 Department of Pediatrics, Maastricht University Medical Centre, 6229 HX Maastricht, The Netherlands; b.delnoy@maastrichtuniversity.nl (B.D.); up202003589@med.up.pt (A.I.C.); 2 Department of Clinical Genetics, Maastricht University Medical Centre+, 6229 HX Maastricht, The Netherlands; 3 GROW-School for Oncology and Developmental Biology, Maastricht University, 6229 HX Maastricht, The Netherlands

**Keywords:** galactosemia type 1, classic galactosemia, galactose 1-phosphate uridylyltransferase (GALT), gene therapy, mRNA therapy, pharmacological chaperones, transcranial alternating current stimulation (tACS), Babble Boot Camp (BBC)

## Abstract

Type I (classic) galactosemia, galactose 1-phosphate uridylyltransferase (GALT)-deficiency is a hereditary disorder of galactose metabolism. The current therapeutic standard of care, a galactose-restricted diet, is effective in treating neonatal complications but is inadequate in preventing burdensome complications. The development of several animal models of classic galactosemia that (partly) mimic the biochemical and clinical phenotypes and the resolution of the crystal structure of GALT have provided important insights; however, precise pathophysiology remains to be elucidated. Novel therapeutic approaches currently being explored focus on several of the pathogenic factors that have been described, aiming to (i) restore GALT activity, (ii) influence the cascade of events and (iii) address the clinical picture. This review attempts to provide an overview on the latest advancements in therapy approaches.

## 1. Introduction

**Figure 1:**
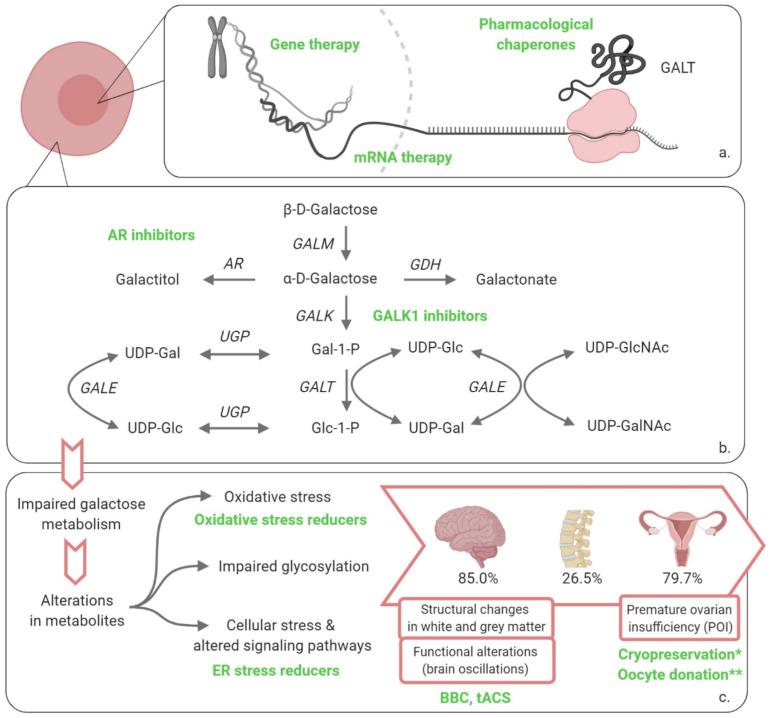
Overview of disorders of galactose metabolism and novel therapeutic approaches recently investigated or under investigation for classic galactosemia. (**a**) Transcription and translation leading to the production of enzyme, therapies restoring enzyme activity; (**b**) Leloir pathway of galactose metabolism and alternative routes of galactose disposal; (**c**) impaired galactose metabolism leading to long-term organ damage. * cryopreservation: more data have become available in the last 5 years; ** oocyte donation: recommendations for CG regarding this procedure have been formulated by a group of experts in the last 5 years. GALM, galactose mutarotase; GALK1, galactokinase 1; Gal-1-P, galactose-1-phosphate; GALT, galactose 1-phosphate uridylyltransferase; Glc-1-P, glucose-1-phosphate; GALE, UDP-galactose 4′-epimerase; UDP-Gal, uridine diphosphate-galactose; UDP-Glc, uridine diphosphate-glucose; UGP, UDP-glucose pyrophosphorylase; UDP-GlcNAc, UDP-*N*-acetylglucosamine; UDP-GalNAc, UDP-*N*-acetylgalactosamine; AR, aldose reductase; GDH, galactose dehydrogenase; ER, endoplasmic reticulum; BBC, Babble Boot Camp; tACS, transcranial alternating current stimulation. Created with BioRender.com.

The galactosemias are a rare group of hereditary disorders of galactose metabolism. To date, four types have been described, each affecting a different step in the main route of galactose disposal: type I, galactose 1-phosphate uridylyltransferase- (GALT, EC 2.7.7.12); type II, galactokinase- (GALK1, EC 2.7.1.6); type III UDP-galactose 4 epimerase- (GALE, EC 5.1.3.2) and the recently described type IV galactose mutarotase- (GALM, EC 5.1.3.3) deficiency, Figure 1 [1,2,3,4,5,6,7,8]. We focus on type I galactosemia and review the latest advancements in therapy approaches.

In type I, classic galactosemia (OMIM 230400), the conversion of α-d-galactose-1-phosphate (Gal-1-P) to α-d-glucose-1-phosphate (Glc-1-P) and uridine diphosphate-galactose (UDP-Gal) is hampered by the severe GALT deficiency. It is an autosomal recessive disorder with a prevalence of 1:16,000 to 1:50,000 live births in Western countries [9,10,11,12,13,14]. The current cornerstone of treatment, a galactose-restricted diet, resolves the neonatal syndrome but fails to prevent burdensome chronic impairments. Most affected patients develop brain impairments (85.0%), primary ovarian insufficiency (79.9%) and a diminished bone mineral density (26.5%) (data based on a large cohort from the Galactosemia network registry (Registry | GalNet (galactosemianetwork.org)). Additionally, data showed that a more favorable outcome among patients is achieved by onset of the diet in the first week of life and detection by newborn screening (NBS). Furthermore, a less strict diet (lactose free without further restrictions) was associated with less neurological complications [6,15]. There is a need for a more adequate treatment to prevent complications.

Classic galactosemia is caused by pathogenic genetic variants in the *GALT* gene, which is located on chromosome 9, leading to a severely diminished enzymatic activity [16]. At present, over 300 genetic variants in the *GALT* gene have been described, the most frequent disease-causing variant among people of European ancestry being the NM_000155.4: c.563A>G genetic variant (p.Gln188Arg) [17]. In addition to classic galactosemia, clinical variants with a higher residual activity are being detected through NBS in which a milder phenotype is anticipated. Structural and functional studies show that in the majority of genetic variants, the dysfunction of the enzyme is due to changes in protein stability and folding [4,5,7,18]. The most frequent genetic variants are NM_000155.4: c.563A>G (p.Gln188Arg); NM_000155.4: c.404C>T (p.Ser135Leu); and NM_000155.4: c.855G>T (p.Lys285Asn) [4,5,7,18].

The pathogenic mechanisms underlying the acute and long-term organ-specific complications of classic galactosemia are complex and remain to be fully elucidated. Currently described contributing factors include accumulation of galactose metabolites (galactitol, galactonate and Gal-1-P) [2,19,20,21,22,23,24,25,26,27,28,29,30], uridine diphosphate (UDP)-hexose alterations and impaired glycosylation [21,25,31,32,33,34,35,36], endoplasmic reticulum (ER) stress with subsequent unfolded protein response (UPR), induction and alteration of signaling pathways [2,3,4,37,38,39,40,41,42], and oxidative stress [4,29,43,44] (Figure 1).

In recent years, a number of animal models (mouse, fruit fly, zebrafish, rat) [24,29,45,46] of classic galactosemia have been developed that mimic, at least partly, the biochemical and clinical phenotypes and complement the cellular models, allowing us to advance our comprehension of the complex playing field of the metabolism of galactose. We have learned that there is different expression of the Leloir pathway components and alternative galactose disposal routes in the different tissues, that combined with specific tissue demands, epigenetic and environmental factors, urge to revisit our understanding. GALT activity levels in affected organs (brain, ovaries) do not seem to differ from the activity levels in organs not affected in classic galactosemia and distinct organ-specific levels of GALT activity have been found [19]. This is reflected in the tissue related differences in relative levels of galactose metabolites (galactitol, galactose and Gal-1-P) in the rat galactosemia model [45], as well as in the nucleotide sugar profiles variation through development and in the different tissues in the zebrafish galactosemia model [31]. Besides, activity differs across different stages in development [19].

The recent elucidation of the crystal structure of human GALT, with a resolution of 1.9 Å, has provided a framework for understanding the molecular consequences of genetic variants and can greatly contribute to the design of therapeutic compounds [5,7]. Novel therapeutic approaches for classic galactosemia currently (defined as: in the last 5 years) being explored aim either to (i) restore GALT activity, (ii) influence the cascade of events or (iii) address the clinical picture. For these strategies to be optimal, characterization of the window of opportunity for treatment is of outmost importance; e.g., neonatal and early infancy or through adolescence or lifelong treatment.

## 2. Potential Therapies

### 2.1. Restoring GALT Activity

Directly restoring GALT activity is the aim of multiple techniques currently being investigated: (i) *GALT* gene therapy, (ii) mRNA therapy and (iii) pharmacological chaperones. Notably, restoring GALT activity up to 10–15% is likely to rescue the phenotype in classic galactosemia. This is suggested by in the so-called “biochemical variant galactosemia” where GALT activity levels between 10% and 15% do not result in clinical disease [47,48].

#### 2.1.1. Gene Therapy

Gene therapy and gene correction techniques aim to permanently compensate for the primary genetic defect [49,50]. In conventional gene therapy, this is achieved by providing patients with the correct coding DNA (cDNA) sequence of the defective gene leading to the expression of normal protein [50,51]. Delivery is accomplished by the use of vectors, either viral or non-viral, containing the genetic sequence of the protein [49,52,53]. In recent decades, significant advances have been made and gene therapy is showing great promise in animal models and clinical trials of inborn errors of metabolism [51,52,53].

In classic galactosemia, viral mediated in vivo gene therapy is currently being investigated using recombinant adeno-associated virus (AAV) vectors [54], which are emerging in the field of gene therapy as very promising [51,52,53]. Rasmussen et al. recently showed, in a pilot study in a galactosemia rat model (*n* = *7*), that neonatal human *GALT* gene therapy with a AAV9 vector via tail vein injection led to GALT restoration in both liver and brain [54]. Relative to wildtype activity, GALT activity levels of 64–595% in liver and 2–42% in brain were found without significant adverse effects. Furthermore galactose metabolites in blood, liver and brain were lowered and a positive effect on cataract was observed [54]. Lai and Loiler expanded the serotypes of the AAV vectors to include AAV8 and AAVrh10, and showed that intravenous administration of AAV8-hGALT and AAVrh10-hGALT resulted in GALT (hGALT) protein expression and decreased levels of Gal-1-P in liver, brain, ovaries and red blood cells of *Galt* knockout mice [55]. In line with this data, Brophy et al. showed that a recombinant AAV vector encoding GALT was able to rescue GALT activity and reduce galactose-induced ER stress in fibroblasts from galactosemic patients [56].

Important future challenges will be focused at safety and efficacy. Safety concerns encompass elicited immune responses by the viral vector capsid proteins and the transgene product, and possible off target effects leading to insertional mutagenesis as well as genomic instability [49,52]. With regard to efficacy, Rasmussen et al. reported differences between liver and brain transgene activity. The authors hypothesized that these differences might stem from preferential perfusion of the liver and limited permeability of the blood–brain barrier and might reflect the differences in activity level of the organs [54]. These findings are consistent with prior reports on AAV9 transgene delivery by intravenous injection [57,58]. The reported studies illustrate the potential of gene therapy for the treatment of GALT-deficient galactosemia.

#### 2.1.2. mRNA Therapy

Exogenous, intracellular delivery of mRNA is emerging as a new class of medicine [59,60,61,62]. Recent advances in mRNA technology have led to improvements in stability and translation by codon optimization and encapsulation within biodegradable delivery systems, preserving mRNA integrity and allowing targeted intracellular delivery [59,60,61,62,63,64,65,66,67,68,69,70]. The required repeated dosing of this therapeutic modality tends to raise concerns about immunogenicity and therapy burden for the patients. As with gene therapy targeting, potential long-term adverse effects therapeutic index and dosing interval of mRNA remain important challenges for the future [60,68,70]. The transient nature of the mRNA requiring repeated dosing to remain effective also bears an advantage, by allowing individual dose titration and cessation of therapy if necessary [60,68,70].

Several preclinical studies evaluating the potential of systemically administered mRNA in metabolic disorders have shown that mRNA provides transient, half-life dependent protein expression, while bearing a low risk of insertional mutagenesis and maintaining dose-responsiveness [67,68,69,71,72,73,74,75]. This therapeutic approach is being explored in classic galactosemia using lipid nanoparticles as delivery system. Balakrishnan et al. recently showed in adult *Galt* knockout mice that systemic administration of LNP-packaged mouse and *hGALT* mRNA resulted in hepatic expression of active, long-lasting GALT enzyme and effectively eliminated galactose 1-phosphate in liver and other peripheral tissues [68]. Using the *galt* knockout zebrafish model, Haskovic et al. also showed successful delivery and translation of functional *hGALT* after single dose injections with LNP-packaged *hGALT* mRNA (*n* = 3) delivered at day 0 and measured on day 5 post fertilization (dpf) [76]. The mRNA approach holds great promise as a potential therapy for classic galactosemia.

#### 2.1.3. Pharmacological Chaperones

Pharmacological chaperones are small molecules that bind specifically to their targets, rescuing specific variant proteins by facilitating intracellular folding and trafficking, increasing cellular stability and activity, and/or preventing premature degradation [4,21]. Pharmacological chaperones present advantageous features: (i) low synthesis cost; (ii) oral availability with broad biodistribution; (iii) low molecular weight that can potentially, allow them to cross the blood–brain barrier (BBB) [77,78,79]. The high occurrence of missense mutations (>60%) [17] and experimental evidence of misfolding and decreased stability of GALT variants makes classic galactosemia highly amenable to this therapeutic approach [18,77,78,79,80,81].

Arginine supplementation, known for its effect as an aggregation inhibitor [82] has been studied as a potential therapeutic approach for classic galactosemia. Despite promising results of this chemical chaperone in a bacterial model [83], in a pilot study with 4 patients carrying the NM_000155.4: c.563A>G (p.Gln188Arg) mutation, arginine did not lead to improved GALT stability nor GALT activity levels as there was no improvement in galactose oxidation capacity [4,84].

Despite being a promising therapeutic approach, its specificity for genetic variants and compound heterozygosity in patient profiles as well as the existence of a broad range of genetic variants complicate the development of pharmacological chaperones as a therapy for all galactosemia patients.

### 2.2. Influence the Cascade of Events

These therapies focus on one of the downstream effects, and as such are less likely to provide treatment for the complete clinical picture of the disease. However, every approach can be helpful by tackling one of the aspects involved in pathophysiology and the combination of treatments could also be an option. The main approaches currently being explored to influence downstream phenomena are inhibitors of galactokinase 1 (GALK1)- and aldose reductase (AR), and endoplasmic reticulum (ER) stress reducers. Oxidative stress reducers have also been proposed in the past, but have not been studied in the last 5 years [43,44,85].

#### 2.2.1. Galactokinase 1 (GALK1) Inhibitors

GALK1 inhibitors were proposed as treatment strategy many years ago [86], and have been thoroughly investigated over the years. The strategy aims to reduce the accumulation of Gal-1-P in GALT deficiency, as Gal-1-P is considered a major key player in the pathogenic mechanism in classic galactosemia [4,12,21]. The milder clinical picture of type II (GALK1 deficiency) galactosemia supported this view although standardized follow-up data are currently lacking [87,88]. Inhibition of GALK1 appears to be an attractive therapeutic target, since GALK1, as member of the GHMP kinase family, is highly substrate specific and no other undesired inhibitions are to be expected [89,90].

High-throughput screenings (HTS) have identified candidate ligands with affinity for GALK1 [12,91,92,93,94]. Promising compounds have been identified and studied to optimize the efficacy and improve the potency. Several compounds have also been tested in cell culture models of patients’ fibroblasts where their efficacy to reduce the biochemical phenotype by lowering Gal-1-P levels has been shown [89,90,91,92,94,95]. The effect of a possible increase in galactitol and galactonate levels remains to be investigated. More research with these compounds beyond cellular models is warranted.

#### 2.2.2. Aldose Reductase (AR) Inhibitors

Aldose reductase (AR) inhibitors target the NADPH-dependent aldose reductase conversion of [2,21,61,96] galactose to galactitol, which cannot be transported across the cell membrane due to its poor diffusivity and cannot be further metabolized, leading to accumulation in cells. This leads to an osmotic phenomenon with subsequent cell swelling and ultimately apoptosis [97,98]. The high expression of aldose reductase in the epithelial cells of the lens leads to the formation of galactosemic cataracts [88,99]. Galactitol involvement in cognitive and neurological symptoms has been suggested [61,100]. Pseudotumor cerebri, or elevated intracranial pressure, has also been reported and is postulated to be the result of accumulation of galactitol in the brain cells leading to cerebral edema [101,102].

AR inhibitors have shown to decrease galactitol levels and prevent cataract formation in hypergalactosemia animal models (rats and dogs) [103,104]. By blocking the galactitol accumulation in rats, the increased osmolarity, UPR and ER stress as well as cell death were prevented [105]. In a hypergalactosemia rat model, Mizisin et al. have, furthermore, shown increased sodium concentrations in endoneurinal fluid in rats that are reversible by AR inhibitors indicating a role for galactitol [106,107,108]. Since the exact role of galactitol in the pathophysiological mechanism of galactosemia remains unknown, other than for cataract, decreasing galactitol levels in other organs might show additional beneficial effects. However, caution needs to be taken since the effect of blocking the polyol pathway that converts galactose into the polyol (sugar alcohol) galactitol, is unknown. An increase in Gal-1-P and galactonate might occur, of which the effects remain to be investigated. AR inhibitors are currently being investigated by the biopharmaceutical company Applied Therapeutics [109].

#### 2.2.3. Endoplasmic Reticulum (ER) Stress Reducers

ER stress has been shown to be involved in the pathology of classic galactosemia, leading to altered signaling pathways, such as the PI3K/Akt pathway [38,41,42]. Balakrishnan et al. have shown that the downregulation of the PI3K/Akt signaling pathway plays an important role in subfertility and cerebellar ataxia in galactosemic mice (*n* = 6) [38]. This indicates that reduced ER stress might be of therapeutic interest for the brain and ovary, suggesting a possible role for eukaryotic initiation factor 2α (eIF2α) inhibitors that reverse the downregulated pathway in fertility preservation and neurological complications. Positive effects of the eIF2α inhibitor salubrinal have been reported in a mouse model of classic galactosemia without detectable adverse effects [39,110]. Moreover, a protective effect on primordial follicle loss, and an increase in fertility, was found [39]. Although salubrinal is an experimental compound, these findings indicate that the downregulation of the PI3K/Akt pathway is a valid potential treatment target in classic galactosemia.

### 2.3. Address the Clinical Picture

#### 2.3.1. Neonatal Period

A galactose-restricted diet resolves the neonatal clinical picture [111]. Newborn screening (NBS) for galactosemia has been implemented in many countries [112,113,114,115,116], and NBS and introduction of diet in the first week of life have shown a beneficial effect [6]. Over the years the strictness of the diet has been questioned and the current recommendation is to eliminate sources of lactose and galactose from dairy products, but permit small amounts of galactose from non-milk sources [111].

#### 2.3.2. Beyond the Neonatal Period

Approaches specifically aimed in tackling the clinical consequences of galactosemia beyond the neonatal period cover the many aspects of the plethora of signs and symptoms associated with GALT-deficiency. In the next paragraphs we touch upon several recent developments regarding (i) fertility preservation, and (ii) neurological and cognitive complications.

##### Fertility Preservation

Primary ovarian insufficiency (POI) with ovarian follicular depletion leading to subfertility is reported in at least 80% of female patients despite a galactose-restricted diet, representing a heavy burden for female patients [6,117,118,119,120,121]. The etiology of ovarian failure and timing of ovarian damage (pre- or postnatal) have not yet been resolved, although studies suggest that follicular depletion has an early initiation [117,120,122,123,124]. Ovarian imaging results show an early onset of ovarian failure (*n* = 14) [125]. Moreover, Mamsen et al. have shown normal follicle morphology and follicle densities in galactosemic girls under 5 years (*n* = 5) [122]. This is supported by the finding of low anti-Mullerian hormone (AMH) levels and low antral follicle counts relative to age-matched controls, suggesting that follicular dysfunction occurs early in life [126,127]. Although spontaneous conception despite POI occurs in classic galactosemia [125,128,129], the chances of pregnancy are severely reduced and fertility preservation options are important. Nowadays, two options are available, cryopreservation and oocyte donation.

The early occurrence of damage supports cryopreservation of ovarian tissue at a very young age as a treatment option [122,125]. Cryopreservation of ovarian tissue has been applied for several years to preserve fertility in patients with (mostly) malignant pathologies that undergo treatments with a detrimental effect on fertility. In recent years, the optimization of cryopreservation strategies and thawing/warming protocols has been achieved, expanding the opportunities for females with various pathologies [130,131]. In classic galactosemia, cryopreservation has to be performed at a very young age, because of the early ovarian damage. Even though this procedure is traditionally associated with a reduction in the ovarian reserve, the technology has improved tremendously over the years [131], and it is now associated with a complication rate of merely 0.2% and an increasing success rate [122,132].

Another approach is intrafamilial oocyte donation (mother-to-daughter or sister-to-sister). In the last five years, a group of experts provided recommendations required to optimally address this option taking into account the professionals as well as the patients and family members views. Topics recommended to be discussed are: family relations, medical impact, patient’s cognitive level, agreements to be made in advance and organization of counseling, disclosure to the child, and need for follow-up [133].

The broad phenotypic spectrum of cognitive and neurological impairments that these female patients might develop make decisions around these complex matters challenging.

##### Neurological and Cognitive Complications

Brain impairments in classic galactosemia, consisting of neurological, cognitive and behavioral complications, are common and are known to have a significant impact on quality of life and general performance [6,134,135,136,137,138,139,140]. Brain impairments have been reported in 85% of patients, including developmental and language delay, neurological complications, language and speech disorders as well as mental and behavioral problems [6]. Structural changes in white and grey matter and functional alterations have been reported [134,135,136,137,138,139].

In addition to the current battery of approaches that are used to help the patients that develop these complications [111], two interesting approaches are currently under study to positively influence language and motor function. One of them is transcranial Alternating Current Stimulation (tACS), a form of non-invasive brain stimulation (NIBS). The rationale behind tACS is to stimulate the naturally occurring rhythmic patterns of electrophysiological activity, also known as brain oscillations, which have been found to be different in classic galactosemia (manuscript in preparation), and modify the oscillations through the delivery of alternating electric currents to the scalp inducing long-term synaptic plasticity [141,142,143,144,145]. Functional networks rely on the synchronization of brain oscillations between the different components of the network, making it an appealing approach for restoring affected functional networks in classic galactosemia [142]. This approach is widely used in cognitive neuroscience, and proven to be efficient in Parkinson and dyslexia and is emerging within the field of psychiatry [141,142,144]. Whether there is proof of concept for this approach is currently under investigation.

The other interesting approach is the Babble Boot Camp (BBC), which is focused on a proactive speech and language intervention program [146]. Current guidelines recommend regular extensive screening for speech and language delay, if children do not meet appropriate levels of speech or language treatment is initiated preferably during the first year of life [111]. However, since speech and language can be regarded as later-developing skill, deficits and signs of delay will only become apparent after an age greater than 24 and 36 months, respectively [111,146]. This approach focuses on a preventive intervention program starting at 2 to 4 months of age and consisting of active parental involvement guided by an expert speech language pathologist. Although the results are preliminary and generalizations cannot be made, a trend to beneficial effects on speech and language development was shown [146].

## 3. Conclusions

In recent years, several therapeutic approaches intended to provide more adequate treatment for classic galactosemia and prevent burdensome long-term complications have been researched. These approaches aim to (i) restore GALT activity, (ii) influence the cascade of events and (iii) address the clinical picture. Gene therapy and mRNA therapy are emerging therapies within the field of medicine, which show great potential in restoring GALT activity levels in animal models. Pharmacological chaperones form an interesting therapeutic approach for rescuing GALT protein, although this approach is genetic variant-specific as opposed to gene therapy or mRNA therapy. Downstream phenomena that are currently being explored aim to reduce ER stress and inhibit GALK1 or AR. Therapeutic strategies specifically tackling the clinical consequences of classic galactosemia are also evolving. For optimal treatment, the window of opportunity is urged to be characterized, defining when treatment is best given for the different complications.

## Data Availability

Not applicable.

## Abbreviations

AAV	Adeno-associated virus
AR	Aldose reductase
BBB	Blood–brain barrier
BBC	Babble Boot Camp
Cas13	CRISPR-associated protein
CRISPR	Clustered regularly interspaced short palindromic repeats
DHEA	Dehydroepiandrosterone
Dpf	Days post fertilization
eIF2α	Eukaryotic initiation factor 2α
ER	Endoplasmic reticulum
Gal-1-P	Galactose-1-phosphate
GALE	UDP-galactose 4-epimerase
GALK1	Galactokinase 1
GALM	Galactose mutarotase
GalNet	Galactosemias Network
GALT	Galactose 1-phosphate uridylyltransferase
GDH	Galactose dehydrogenase
GHMP	Galacto-, homoserine-, mevalonate- and phosphomevalonate kinase
Glc-1-P	Glucose-1-phosphate
hGALT	human GALT
HTS	High throughput screening
LNP	Lipid nanoparticle
NADPH	Nicotinamide adenine dinucleotide phosphate
NBS	Newborn screening
NIBS	Non-invasive brain stimulation
PI3K/Akt	Phosphoinositide 3-kinase/protein kinase B
POI	Primary ovarian insufficiency
ROS	Reactive oxygen species
tACS	Transcranial alternating current stimulation
UGP	UDP-glucose pyrophosphorylase
UDP	Uridine diphosphate
UDP-Gal	Uridine diphosphate-galactose
UDP-Glc	Uridine diphosphate-glucose
UDP-GalNAc	UDP-*N*-acetylgalactosamine
UDP-GlcNAc	UDP-*N*-acetylglucosamine
UPR	Unfolded protein response
